# Antibacterial Stability of Novel Nisin/Carboxylic Curdlan Complexes

**DOI:** 10.3390/foods14234007

**Published:** 2025-11-22

**Authors:** Long-Qing Li, Yun-Bo Yu, Shu-Yan Zhang, Zheng-Cai Liu, Le-Yi Pan, Tong-Xin Liang, Ming-Yu Jin, Ya-Hui Yu, Jing-Kun Yan

**Affiliations:** 1 Engineering Research Center of Health Food Design & Nutrition Regulation, Dongguan Key Laboratory of Typical Food Precision Design, China National Light Industry Key Laboratory of Healthy Food Development and Nutrition Regulation, School of Life and Health Technology, Dongguan University of Technology, Dongguan 523808, China; lilongqing21@126.com (L.-Q.L.); zhangsy@dgut.edu.cn (S.-Y.Z.); lzzcc0704@163.com (Z.-C.L.); leyipan@163.com (L.-Y.P.); m13676152162@163.com (T.-X.L.); jinmy92@163.com (M.-Y.J.); yuyahui@dgut.edu.cn (Y.-H.Y.); 2 School of Food & Biological Engineering, Jiangsu University, Zhenjiang 212013, China; 2281007063@163.com

**Keywords:** nisin, carboxylic curdlan, complex coacervation, stability, antibacterial activity

## Abstract

Nisin is a widely used natural antibacterial peptide in the food industry. However, its instability limits its practical application in food preservation. In this study, the antibacterial activity of previously built nisin/carboxylic curdlan (C6-Cc) complexes were tested against Gram-positive bacteria: Staphylococcus aureus. The results revealed that when the mass ratio of C6-Cc to nisin was 1:8 (C6-Cc: nisin, *w*/*w*), the nisin/C6-Cc complexes exhibited strong antibacterial stability over the pH range of 1–4, with inhibition zone diameters ranging from 11.5 to 13.0 mm and a minimum inhibitory concentration (MIC) of 0.71 mg/mL. Notably, the complexes also maintained good thermal stability even at 121 °C. Furthermore, the complexes with a mass ratio of 1:8 (*w*/*w*) showed superior physicochemical stability under low-pH conditions (pH < 5) within the salt concentration of 0–100 mM, and the stable temperature ranged from 25 to 121 °C. Twenty-eight days of storage had no significant impact on its antibacterial or physicochemical stability. These findings suggest that the nisin/C6-Cc complexes will have broad application prospects in food bio-preservation.

## 1. Introduction

With the growing awareness of food safety, extending shelf life and preventing food spoilage caused by microorganisms have emerged as pressing issues in the field of food science. The constant consumer demand for safe and healthy food is driving the development of bio-preservation techniques [1,2]. Among natural food preservatives, nisin is an antibacterial peptide produced by *Lactococcus lactis* spp., which is the only officially recognized non-toxic bacteriocin with an inhibitory effect on the growth of a broad range of Gram-positive bacteria and spores. Nisin is a cationic peptide consisting of 34 amino acids and contains four positive charges [3]. As the most extensively studied elongated type-A lantibiotic [4], nisin has been proved to reduce the reliance of sterilization processing while improving the nutrition and flavor of food during the food production process. Nisin has been widely used in dairy products, in meat products, in fruit juices [5], and in canned foods to extend shelf life under high temperatures [6,7]. However, as a hydrophobic peptide, nisin is often affected by various factors in the food industry, such as salts, pH, processing temperatures, and even enzymes or fats. These influences can lead to poor stability and weakened antibacterial efficiency, thereby greatly limiting its practical application in food preservation [3].

Microencapsulation technology is one of the most effective strategies to make up for the defects of the unstable antibacterial effect of nisin to improve the proteolytic stability of nisin [8]. Complex coacervation, a self-aggregation phenomenon driven by electrostatic interaction between two or more charged macromolecules [9], is one of the simplest and most sustainable microencapsulation methods to improve antibacterial activity retention under stress conditions, which is usually defined as antibacterial stability. Complex coacervation has been exploited for the design of soft formulations with various application, including food and medicine technology [10,11]. As a cationic antimicrobial peptide, nisin can form microcapsules with food-borne polyanionic polysaccharides through complex coacervation to achieve nisin embedding, controlled release, and antibacterial stability [12]. Moreover, researchers have used complex coacervation to coat the nisin/polyanionic polysaccharide complex with a cationic biopolymer protective layer to construct complex coacervation microcapsules [13,14,15,16], thereby reducing the environmental sensitivity of microcapsules and enhancing the stability of nano-delivery systems. Thus, complex coacervation has become an interesting research area in the field of nisin microencapsulation [17].

Curdlan is a water-insoluble β-glucan produced by *Alcaligenes faecalis*, which can form thermally reversible or irreversible gels depending on the applied heating temperature. Due to its specific gel properties and unique molecular structure, it has a wide range of applications and development value in food, medicine, and healthcare [18]. However, curdlan also exhibits a non-negligible limitation of inert solubility both in organic and aqueous solvents [19]. To further expand the application of curdlan, regioselective oxidations of C-6 primary hydroxyls of polysaccharides to carboxylate groups have been developed, in which curdlan can be completely oxidized at the C-6 position to obtain carboxylic curdlan (C6-Cc) [20], which is ideal for forming complexes with cationic nisin via electrostatic interactions.

Although previous studies have confirmed that complex coacervation can stabilize nisin [17], most of the mechanism interpretation lacks systematic verification of actual food processing conditions. Our recent study has reported the use of C6-Cc for controllable encapsulation of nisin, where the key coacervating mechanism of nisin/C6-Cc complexes was strong electrostatic interactions followed by hydrophobic interactions and hydrogen bonding. Moreover, the nisin/C6-Cc complexes were also proved to have good viscoelastic properties and predominant elasticity [21], while the antibacterial and physicochemical stability of nisin/C6-Cc complexes for food application remain unexplored. Therefore, this study will further explore the advantages of physicochemical and antibacterial stability of the nisin/C6-Cc complexes under various conditions (including the changes in pH, ion strength, and temperatures), and the relative antibacterial properties will also be analyzed as well. These findings will provide experimental evidence and theoretical support for the potential application of nisin/C6-Cc complexes in food bio-preservation.

## 2. Materials and Methods

### 2.1. Materials and Chemicals

Nisin (900 IU/mg) was purchased from Shanghai Yuanye Biological Co., Ltd. (Shanghai, China). C6-Cc was obtained according to our previous study, where the curdlan was successfully derivatized with an oxidation degree of approximately 96% [22] (Qiu et al., 2018), and the nisin/C6-Cc complex samples with different mass ratios were well prepared with reference to Jin et al. (2025). The *Staphylococcus aureus* (ATCC6538) strain was purchased from Zhejiang Institute of Microbiology (Hangzhou, China), and Luria–Bertani (LB) liquid and solid media were purchased from Sangon Biotech Shanghai Co., Ltd. (Shanghai, China). The other reagents are all analytical reagents, which were purchased from the National Pharmaceutical Group Chemical Reagents Co., Ltd. (Shanghai, China), and distilled water was used in all experiments.

### 2.2. Antibacterial Activity of Nisin/C6-Cc Complexes

#### 2.2.1. Inhibition Zone Assay

In this study, *S. aureus* ATCC6538 was used for the antibacterial activity assessment, and the bacterial inhibition zone was determined by the agar diffusion method according to Meng et al. (2018) [23]. Briefly, the *S. aureus* was cultured at 37 °C using a constant temperature shaker for 24 h. The concentration of *S. aureus* bacterial suspension was then adjusted to 10^6^ CFU/mL, and 100 μL of suspension was drawn on the surface of each LB solid agar plate. After the medium was completely solidified, a 5 mm hole was poked at an appropriate position, and 20 μL of the nisin/C6-Cc complex solution under different treatment conditions was added to the hole. The solution was incubated at 37 °C for 12 h. For thermal stability analysis, all mixtures of the three mass ratios were heated at 20, 40, 60, 80, 100, and 121 °C, respectively. The diameter of the inhibition zone was measured by the cross method by using a measuring tool to draw two perpendicular lines (forming a cross) at the bottom of the culture dish and taking the cross point as the starting point of the colony diameter. After the colony grew to a certain size, the diameter of the colony on the two straight lines was measured using a microscope or visual method, the data were recorded, and the average value was taken for further analysis.

#### 2.2.2. Determination of Minimum Inhibitory Concentration (MIC)

MIC is defined as the minimum concentration required for complete inhibition of bacterial growth. The MIC of the nisin/C6-Cc complexes under different treatment conditions was determined using 96-well microtiter plate assays and the double dilution method as described previously with minor modifications [24]. Briefly, the bacterial liquid was adjusted to 10^6^ CFU/mL in the mid-log growth stage with fresh LB medium, and 100 μL of the standardized bacterial suspension was inoculated into each plate well. The nisin/C6-Cc complex solution with different treatments was added to 100 μL of the first well of the 96-well plate, and 100 μL of LB liquid medium was added to the first well. Then, 100 μL of the mixed solution was drawn into the second well, and 100 μL of LB liquid medium was added to the second well for the next mixture. The procedure was repeated ten times. Moreover, a well containing 200 μL of *S. aureus* suspension (10^6^ CFU/mL) was settled as a positive control and 200 μL of LB liquid medium as a negative control. The MIC values were measured at 0 and 30 days of storage. Each test was repeated at least three times, all three replicates produced identical concentrations, and the entire operation was completed on a sterile ultra-clean bench. The plate was placed in an incubator at 37 °C for 16–20 h. The results were recorded following optical density measurement as the minimum concentration of aseptic growth was the MIC value.

### 2.3. Dynamic Light Scattering (DLS) Analysis

The particle size, Zeta potential, and polydispersity index (PDI) value of the nisin/C6-Cc complex solutions were determined by a laser particle analyzer (LitesizerTM500, Anton Paar, Graz, Austria) at 25 °C. The nisin/C6-Cc complex solutions with different mass ratios of 1:4, 1:8, and 1:14 (C6-Cc: nisin, *w*/*w*) were prepared according to Jin et al. (2025) for measurement. The laser source power was set at 40 mW, the detection angle was 175°, and the detection wavelength was 658 nm.

### 2.4. Physical Stability of Nisin/C6-Cc Complexes

#### 2.4.1. Thermal Stability

To evaluate the thermal stability of complexes, the nisin/C6-Cc complexes with mass ratios of 1:4, 1:8, and 1:14 (C6-Cc: nisin, *w*/*w*) were prepared according to Jin et al. (2025) [21] and studied under different temperature conditions. Briefly, all mixtures of the three mass ratios were heated at 20, 40, 60, 80, and 100 °C for 15 min under water bath conditions and then cooled to 25 °C. Then, the particle size, Zeta potential, PDI, and antibacterial activity were measured, respectively.

#### 2.4.2. pH Stability

According to the antibacterial activity and thermal stability results, the nisin/C6-Cc complexes with a mass ratio of 1:8 (C6-Cc: nisin, *w*/*w*) were selected for further stability studies. Briefly, the nisin/C6-Cc complexes were prepared as previously reported [21]. The pH value of the nisin/C6-Cc complexes was adjusted by adding 0.1 M NaOH solution and 0.1 M HCl solution from 1 to 7, respectively. Then, the particle size, PDI, and Zeta potential of the nisin solution, C6-Cc, and nisin/C6-Cc complex aqueous solutions were measured by DLS analysis using a laser particle size analyzer (LitesizerTM500, Anton Paar, Austria), respectively. The laser source power was 40 mW, and the detection angle was 175°.

#### 2.4.3. Salt Ion Stability

For analysis of salt ion stability, different concentrations of sodium chloride (0, 50, 100, 150, and 200 mM) were added to the solution of nisin/C6-Cc complexes with a mass ratio of 1:8 (C6-Cc: nisin, *w*/*w*), respectively. The mixture was stirred evenly, and its particle size, Zeta potential, and PDI were measured under different ionic strengths, respectively.

#### 2.4.4. Storage Stability

For the storage stability analysis, the nisin/C6-Cc complexes with a mass ratio of 1:8 (C6-Cc: nisin, *w*/*w*) were packaged in sealed glass bottles, stored in an incubator at 25 °C without shaking and 4 °C in a refrigerator for 28 days, respectively. The particle size, Zeta potential, PDI, and antibacterial activity were measured at 0, 7, 14, 21, and 28 days of storage, respectively.

### 2.5. Statistical Analysis

All determinations were performed at least three times, and values are expressed as the mean ± standard deviation (SD). One-way analysis of variance (ANOVA) was carried out using Origin software (version 8.0, Origin Lab Corp., Northampton, MA, USA) to determine the differences between samples. Tukey’s Honest Significant Difference (HSD) post hoc test was conducted to identify pairwise significant differences between individual sample groups. A statistical significance threshold was defined as *p* < 0.05, and all reported differences were considered significant only when the *p*-value was lower than this threshold.

## 3. Results and Discussion

### 3.1. Antibacterial Stability of Nisin/C6-Cc Complexes

#### 3.1.1. Thermal Effects of Antibacterial Activity of Nisin/C6-Cc Complexes

**Figure 1:**
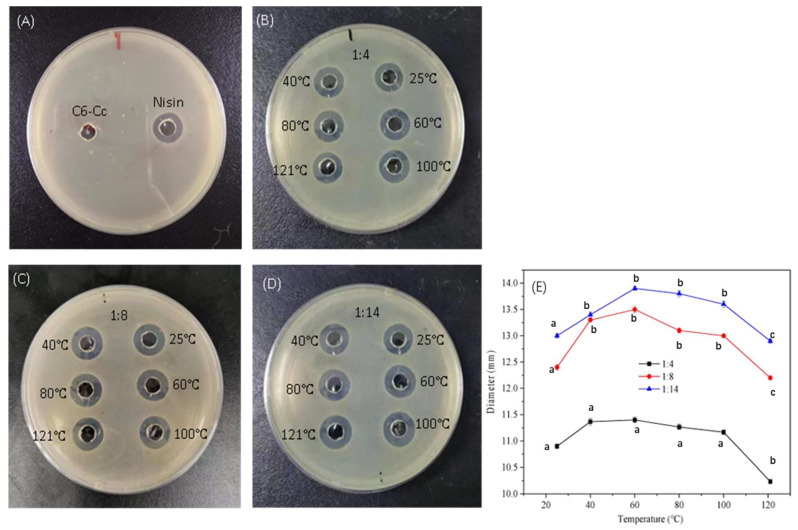
Pictures of nisin/C6-Cc complex inhibition zone at different temperatures ((**A**) is pictures of C6-Cc and nisin at 2 mg/mL; (**B**), (**C**) and (**D**) are mass ratios of 1:4, 1:8, and 1:14, respectively, *w*/*w*) and the diameter of inhibition zone (**E**). Lowercase letters within the same row indicate significant differences according to the ANOVA test (*p* < 0.05).

Since nisin is commonly used as a natural food preservative in beverages and dairy products, it is of great significance to discuss the thermo effect on the antibacterial properties of nisin/C6-Cc complexes with various mass ratios (1:4, 1:8, and 1:14 *w*/*w*). As shown in Figure 1A, there was no inhibition zone in the sample of C6-Cc solution, which indicated that C6-Cc had no antibacterial effect on *S. aureus*, and the formation of the inhibition zone in nisin/C6-Cc complexes was mainly due to the presence of nisin. In Figure 1B–D, the diameter of the inhibition zone of the nisin/C6-Cc complexes gradually increased with increasing nisin mass ratio, indicating the antibacterial activity was positively correlated with the amount of nisin. With increases in temperature, the diameter of the inhibition zone increased first and then dropped, indicating the antibacterial activity was affected by the temperature and the diameter of the inhibition zone of three nisin/C6-Cc complexes all reached their maxima at 60 °C. In addition, the nisin/C6-Cc complexes still have strong inhibition zone diameters with C6-Cc to nisin mass ratios of 1:8 and 1:14 (12.2 mm and 12.9 mm, respectively) after heating at 121 °C for 15 min (Figure 1C–E), indicating good thermal stability of the complexes. Wang et al. (2023) [25] once proposed that free nisin cannot form an inhibition zone and loses its antibacterial activity after sterilization at 121 °C for 5 min, but microencapsulated nisin could produce an inhibition zone against *S. aureus* and the antibacterial rate of encapsulated nisin was more stable than that of free nisin after treatment at 120 °C for 20 min. These results prove that microcapsule treatment can maintain the antibacterial activity of nisin with high thermal stability. In this study, the nisin/C6-Cc complexes with mass ratios of 1:8 and 1:14 (*w*/*w*) both maintain good antibacterial activity after treatment at 121 °C for 15 min, revealing that composite coacervation can effectively improve the antibacterial stability of nisin under high-temperature conditions.

Nisin has a strong resistance to the growth and reproduction of Gram-positive bacteria and spore microorganisms. However, spore microorganisms generally need to be inactivated at a high temperature of 121 °C. Therefore, our research also investigated the effect of temperature on the antibacterial stability of nisin/C6-Cc complexes. Table 1 showed the MIC variation of nisin/C6-Cc complexes at different temperatures. The nisin/C6-Cc complexes at the C6-Cc to nisin mass ratios of 1:8 (*w*/*w*) have stable antibacterial activity even at 121 °C (Figure 1), indicating the antibacterial activity of the nisin/C6-Cc complex at this specific ratio was not compromised by high temperatures. According to Schmitt et al. [26], this may be due to the charge matching degree of C6-Cc and nisin being the highest at the ratio of 1:8 and the formed complex crosslinking network being denser at high temperature, leading to strong thermal stability of the complexes itself, which can well protect the antibacterial active site of nisin. In this case, nisin can maintain strong antibacterial activity and retain its inactivation effect on spore microorganisms after microencapsulation through complex coacervation with C6-Cc. It is worth noting that when the C6-Cc to nisin mass ratio is 1:14 (*w*/*w*), the MIC value of the complexes only maintains its high antibacterial activity at 25 °C, while at 40–121 °C, the MIC value is 1.03 mg/mL, which was 1.2–1.5 times higher than the MIC values of the 1:4 and 1:8 ratio complexes at the same temperature range (Table 1). This difference suggests that not all nisin/C6-Cc formulations exhibit equivalent thermal stability. A potential explanation for this is that the lower C6-Cc content in the 1:14 ratio (compared to 1:8) leads to a less dense crosslinking network, which is insufficient to protect nisin’s active sites from thermal degradation. Thus, the nisin/C6-Cc complex with a C6-Cc-to-nisin mass ratio of 1:8 (*w*/*w*) demonstrates robust thermal stability and antibacterial activity under high temperatures, making it a suitable candidate for further studies.

#### 3.1.2. Storage Stability of Antibacterial Activity of Nisin/C6-Cc Complexes

**Figure 2:**
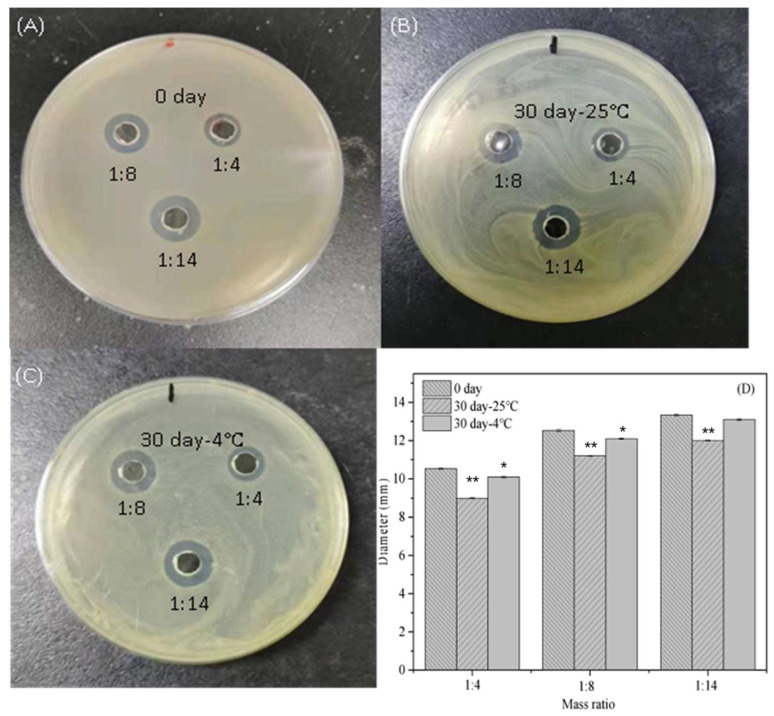
Pictures of the inhibition zone of nisin/C6-Cc complex under different storage conditions ((**A**) 0 days, (**B**) 30 days at 25 °C, (**C**) 30 days at 4 °C) and the influence of the diameter of the inhibition zone (**D**). (* *p* < 0.05; ** *p* < 0.001; 0 day as control, comparing within groups).

The storage stability was also critical to the antibacterial activity of nisin/C6-Cc complexes. As shown in Figure 2, the antibacterial activity of nisin/C6-Cc complexes was positively correlated with an increasing C6-Cc to nisin mass ratio. The freshly prepared nisin/C6-Cc complexes had strong antibacterial activity, and the diameter of the inhibition zone of the nisin/C6-Cc complexes were still close to the fresh prepared samples after storage at 4 °C for 30 days, while the antibacterial activity decreased significantly after storage at 25 °C for 30 days (*p* < 0.05). However, there was no significant difference on antibacterial activity between the nisin/C6-Cc complexes with mass ratios of 1:8 and 1:14 (*w*/*w*).

Moreover, Table 2 revealed that the MIC values of the nisin/C6-Cc complexes with C6-Cc to nisin mass ratios of 1:4 and 1:8 (*w*/*w*) have no difference from those of the freshly prepared ones after storage at 25 °C and 4 °C for 30 days, respectively, indicating these two nisin/C6-Cc complexes have strong storage stability. When the C6-Cc to nisin mass ratio was 1:14 (*w*/*w*), the MIC value increased from 0.51 mg/mL to 1.03 mg/mL after 30 days of storage, and its antibacterial activity also reduced. This may due to the large particle size and poor stability of the complex aggregates at the mass ratio of 1:14 (*w*/*w*), where the antibacterial activity of nisin bound to C6-Cc was also limited to a certain extent. Collectively, these results indicated that the antibacterial activity of nisin/C6-Cc complexes could be well maintained at 4 °C, and the nisin/C6-Cc complexes with a mass ratio of 1:8 (C6-Cc: nisin, *w*/*w*) are suitable for further studies.

#### 3.1.3. pH Effects of Antibacterial Activity of Nisin/C6-Cc Complexes

**Figure 3:**
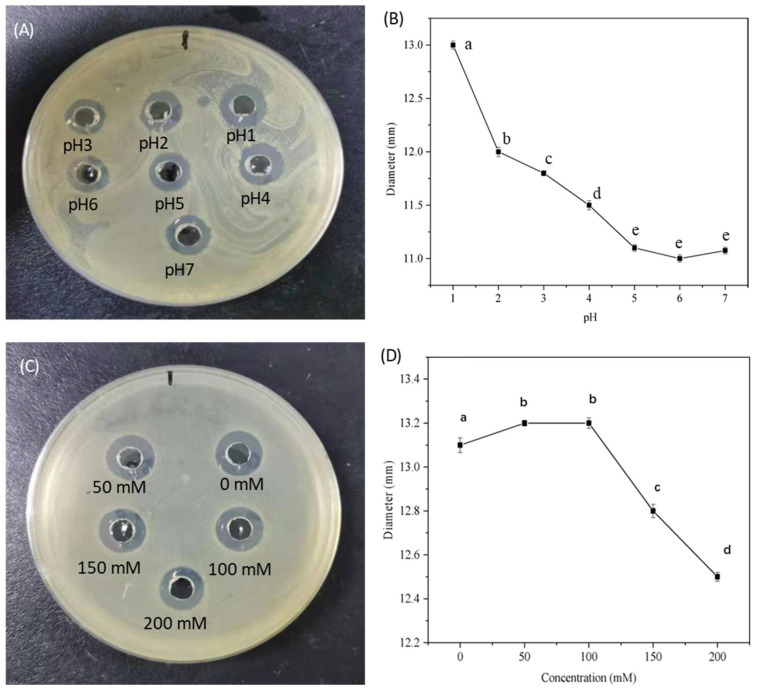
Inhibition zone of nisin/C6-Cc complex at different pH (**A**) and inhibition zone diameter (**B**), and the inhibition zone at different salt ion concentrations (**C**) with the diameter (**D**), respectively. Lowercase letters within the same row indicate significant differences according to the ANOVA test (*p* < 0.05).

The pH effect on the antibacterial activity of selected nisin/C6-Cc complexes with a mass ratio of 1:8 (C6-Cc: nisin, *w*/*w*) were further investigated. Briefly, with the increase in pH, the inhibition zone of nisin/C6-Cc complexes against *S. aureus* was smaller (Figure 3A,B). The inhibition zone was as high as 13.0 mm at the pH of 1.0, while the diameter of the inhibition zone sharply decreased to around 11.0 mm from pH of 5, indicating that the higher pH could have negative effects on its antibacterial activity. This result was consistent with the results of Khelissa et al. (2021) [6], where the lower pH value of the solution could provide a better dissolution environment of nisin, leading to more release of active groups and bonding sites to interact with Gram-positive bacteria, which leads to the enhancement of antibacterial activity. Previous studies have proved that *S. aureus*, a common food-borne pathogen that poses a serious threat to human health [27], had a high prevalence (~40%) in retail meat samples in several Europe countries [28]. Therefore, natural bacteriocins with high efficacy have gradually become a crucial tool for the control of food-borne pathogen [29]. Considering that pH 1–2 is more acidic for most food matrices, the possible practical pH value of the complex could be applied in the range from 3 to 4.

The MIC values of nisin/C6-Cc complexes at C6-Cc to nisin mass ratio of 1:8 (*w*/*w*) we also determined by double dilution method in 96-well plates. In Table 3, the MIC value of nisin alone was only 0.31 mg/mL, indicated the nisin had strong antibacterial activity against *S. aureus*, while C6-Cc had no antibacterial activity, which was consistent with the results of Figure 1. With the increase in the pH of the nisin/C6-Cc solution, the MIC value remained at a low concentration of 0.71 mg/mL between pH 1 and 4. Then, the MIC value increased to 1.43 mg/mL from pH 5 to 7, indicating the weakened antibacterial activity of nisin/C6-Cc complexes at higher pH values. The core mechanism of this result is the regulation of pH on the interface charge and nisin structure of the complex. At low pH (1–4), the carboxyl group (-COOH) of C6-Cc has a low degree of protonation and retains more negative charges. Strong electrostatic interaction with positively charged nisin could combine with hydrophobic interaction and hydrogen bonding to construct a dense cross-linked network to avoid the dissociation of nisin. At the same time, the low pH environment can stabilize the β-sheet structure of nisin [7], so that its antibacterial active sites (such as dehydroalanine residues) are intact, thereby efficiently binding to bacterial cell membranes. When pH > 5, nisin is close to its isoelectric point pI ≈ 8.8 [25], where the positive charge is reduced, the electrostatic interaction with C6-Cc is weakened, leading to dissociated of complex. The structure of nisin was denatured due to the exposed and inactivated active site, which eventually led to a decrease in antibacterial ability. This result may solve the problem that free nisin is easy to deactivate and difficult to retain in acidic beverages [5,30].

#### 3.1.4. Effects of Ionic Strength on Antibacterial Activity of Nisin/C6-Cc Complexes

Figure 3C, D further showed the antibacterial activity of the nisin/C6-Cc complex solution with a C6-Cc to nisin mass ratio of 1:8 (*w*/*w*) at different salt ion concentrations. The diameter of the inhibition zone was first slightly increased and then decreased with the enhancement of ionic strength, which is consistent with the effect of ionic strength on the stability of the ionic strength, the results of which are shown in the following Table 3. The possible mechanism could be the charge shielding effect of salt ions and the stabilization effect at low concentrations. At high ionic strength (>100 mM), Na^+^/Cl^−^ shields the negative charge of C6-Cc and the positive charge of nisin and weakens the electrostatic interaction, leading to the slight dissociation of some complex particles. However, due to the high carboxyl content of C6-Cc, the core cross-linked structure could still be maintained, the antibacterial activity did not decrease significantly. However, at a low ionic strength of 50 mM, salt ions weakened the repulsive force between the C6-Cc and nisin molecules [31], promoted tighter binding between C6-Cc and nisin, and slightly increased the diameter of the inhibition zone to 13.2 mm (Figure 3D). This result is complementary to the pH regulation results, where the pH affects the stability of the complex by changing the molecular charge properties, while ionic strength fine-tunes the structure by adjusting the degree of charge shielding, and the stability of the complex at 0–200 mM ionic strength is significantly better than the existing nisin–polysaccharides complexes [32], further demonstrating that its advantages of salt tolerance may be suitable for salted food application in terms of stability and strong antibacterial activity.

### 3.2. Thermo Stability of Nisin/C6-Cc Complexes

**Figure 4:**
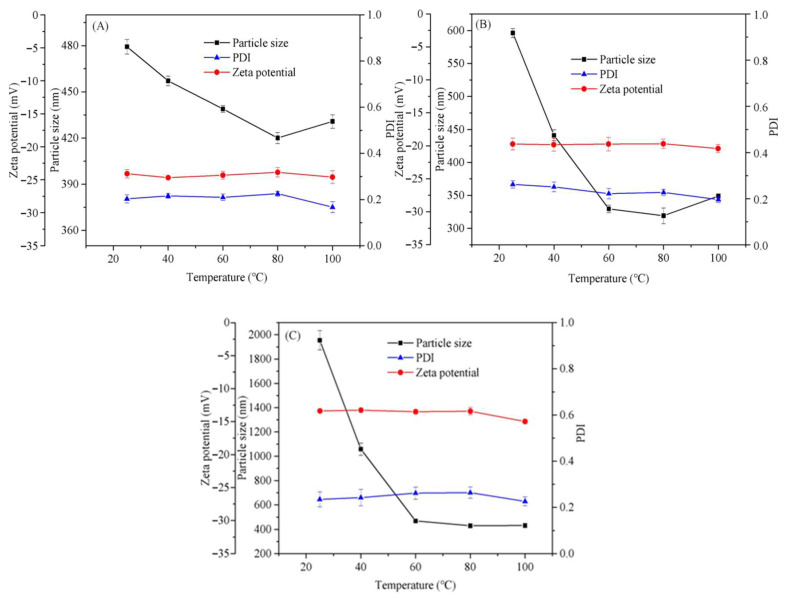
Effect of thermal treatment on the stability of nisin/C6-Cc complex coacervate ((**A**), (**B**), and (**C**) are, respectively, 1:4, 1:8, and 1:14 mass ratio).

Since the nisin/C6-Cc complexes have strong antibacterial activity, it is necessary to study their physicochemical stability. Temperature is a crucial factor affecting the formation of polysaccharide–protein complexes, where the hydrophobic interaction dominates the reaction [26]. In Figure 4, it can be observed that the Zeta potential of nisin/C6-Cc complexes rarely changed with increasing temperature and only showed a slightly decrease at 100 °C, indicating that the small decrease may be due to two reversible physical changes in the weak effect at 100 °C on the dissociation state of the C6-Cc carboxyl group. C6-Cc is a carboxyl polysaccharide with a high oxidation degree, and the dissociation equilibrium of carboxyl (-COO) on its surface is sensitive to temperature [19]. In this study, this dissociation fluctuation may slightly reduce the net negative charge on the surface of the complex, showing a slight decrease in zeta potential. However, the complex still maintains a small particle size (319.2–479.3 nm) and low PDI (<0.3) at 100 °C. The weak change in carboxyl dissociation does not break the core electrostatic balance between C6-Cc negative charge and nisin positive charge, and the cross-linked network of the complex is still intact. On the other hand, the hydration layer on the surface of the composite induced by high temperature is slightly desorbed. Schmitt et al. (1998) [26] pointed out that there is a dynamic hydration layer on the surface of the protein–polysaccharide complex, and high temperature will promote the desorption of some weakly bound water molecules, resulting in local fine-tuning of the charge distribution on the surface of the complex. This adjustment only affects the interface electric double layer thickness during zeta potential detection, rather than the intrinsic strength of intermolecular electrostatic interactions. In this study, the particle size of the composite did not increase significantly at 100 °C (compared with 596.2 nm at 25 °C, it only decreased to 479.3 nm), and the PDI was always <0.3, which proved that the desorption of the hydration layer did not cause particle aggregation, and the small decrease in zeta potential was a reversible physical phenomenon, rather than a signal of impaired stability.

In the range of 25–121 °C, the particle size of nisin/C6-Cc complexes sharply reduced from 25 °C to 80 °C and then slightly increased at 121 °C, and the complexes achieve the largest particle size at 25 °C. The hydrophobic interaction is weakened and the hydrogen bonding is enhanced at lower temperatures, which is beneficial to the complex coacervation reaction of nisin and C6-Cc. The possible explanation might be that low temperature is conducive to the formation of hydrogen bonds in the complexes, while a high temperature may denature the protein and expose more active sites, promoting the formation of complexes [33]. The complex coacervation reaction is the strongest at the C6-Cc to nisin mass ratio of 1:8 (*w*/*w*) in which complexes with maximum particle size was formed. In Figure 4C, the particle size of the complexes is 1954.8 nm at the mass ratio of 1:14, and the particle sizes at mass ratios of 1:4 and 1:8 are 497.3 nm and 596.2 nm, respectively. Under the larger mass ratio, more nisin could react with C6-Cc to form a larger complex coacervation. In contrast, with the temperature raised to 40 °C, the hydrophobic effect of the complexes is enhanced, where the molecules in the system are rearranged [34] to form smaller complexes with smaller particle size. In Figure 4, the particle size of the nisin/C6-Cc complexes are at their minimum at 80 °C, and the particle size of the complexes showed a sharp decrease with increasing nisin ratio. Although the particle size slightly fluctuated at 121 °C, the overall results still revealed that the system has good particle stability at 60–121 °C. Meanwhile, when the mass ratio was 1:14, the complexes maintained a smaller particle size of 430 nm within 60–100 °C, which may be due to the denaturation and the structure variation in the peptide at high temperatures, leading to peptide extension for a better combination with C6-Cc. However, when the mass ratios were 1:4 and 1:8, the particle sizes of the complexes were in the range of 420.0–479.3 nm and 319.2–596.2 nm, respectively, and the PDI values were below 0.3, showing good uniformity. The above results all prove that the complexes showed better stability among 25–121 °C at the mass ratios of 1:4 and 1:8. Based on the abovementioned results, nisin/C6-Cc with a mass ratio of 1:8 may be more suitable for application in dairy products, which is consistent with abovementioned antibacterial activity study in Figure 1.

### 3.3. pH Stability of Nisin/C6-Cc Complexes

**Figure 5:**
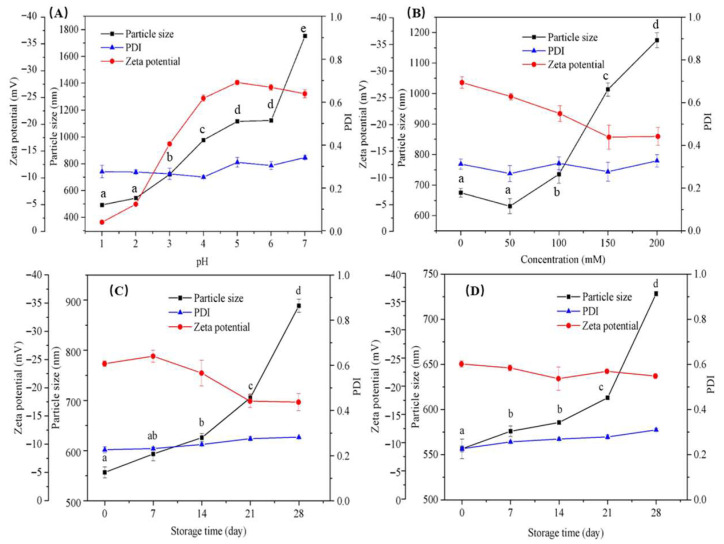
Effect of pH (**A**), ion strength (**B**), and storage condition ((**C**)—25 °C; (**D**)—4 °C) on the stability of nisin/C6-Cc complex (the figure shows the mass ratio of 1:8, *w*/*w*). Lowercase letters within the same row indicate significant differences according to the ANOVA test (*p* < 0.05).

Figure 5A showed the effects of different pH values on the particle size, Zeta potential, and PDI values of selected nisin/C6-Cc complexes at the C6-Cc-nisin mass ratios of 1:8 (*w*/*w*). With the increase in pH, the particle size of the complexes also gradually increased. When the mass ratio of nisin/C6-Cc was 1:8, the pH_opt_ (the pH for the turbidity of the mixed system reaches the maximum value) of nisin/C6-Cc complexes was 3.0 [21], and the maximum complexes was formed in the solution system. The particle size of the solution increased sharply from 543.4 nm to 720.8 nm. At the pH of 3.0, the surface of nisin in solution is positively charged, and the surface of C6-Cc is negatively charged, where the two components have the strongest electrostatic attraction force, leading to a tight combination with insoluble complexes. With constant increasing pH values near the isoelectric point of nisin (pH = 8.8) [35], the decrease in nisin charge will cause looser binding of the nisin/C6-Cc complexes, which leads to larger particle sizes. When the pH of the system is greater than 5, the particle size of the nisin/C6-Cc complexes is positively correlated with the pH. Based on our previous study [21], it could be explained that both nisin and C6-Cc are negatively charged at this pH condition. As the pH becomes more acidic, the anionic charge of C6-Cc decreases and the cationic charge of nisin increases. Due to the influence of electrostatic repulsion, a small amount of heat-induced aggregate dissociation occurs in the system [36], while the free nisin is unstable at a high pH and its particle size is relatively large. The particle size of the individual C6-Cc solution also increased with increasing pH. Therefore, the higher pH of the complexes could cause a larger particle size, which leads to an unstable trend of the system. In contrast, the particle size of nisin/C6-Cc complexes is smaller at lower pH (pH < 5), and its PDI value is maintained between 0.25 and 0.28, where the system is in a relatively uniform state. This result implied that the maximum electrostatic attraction was achieved and coacervates with the strongest gel-like network structure were formed, which may be dominated by elasticity due to strong electrostatic interactions [37]. However, the PDI value of the complex solution exceeds 0.3 when the pH is over 5, suggesting that the complexes particles over this pH are unstable. Considering the particle size, PDI value, and Zeta potential, nisin/C6-Cc complexes are more stable at low pH (pH < 5) and can be applied to acidic beverages and canned foods.

### 3.4. Ionic Stability of Nisin/C6-Cc Complexes

Nisin has been applied as a preservative in various food products, in which salt ions are inevitable. Therefore, it is of great practical significance to explore the ionic stability of nisin/C6-Cc complexes. As shown in Figure 5B, the particle size of the nisin/C6-Cc complexes increases with increasing ionic strength. This may be because of the ‘charge shielding’ effect of ions interfering with the complex coacervation reaction of peptides and polysaccharides, causing the dissociation of some particles in the system [36]. However, it is worth noting that when the ionic strength is 50 mM, the particle size of the system decreases slightly from 674.8 nm to 630.7 nm. In fact, previous studies have shown that high concentrations of salt ions could inhibit the association of electrostatic attraction between proteins and polysaccharides, while low concentrations of salt ions could promote this electrostatic attraction [38,39]. Wang et al. (2007) [31] once suggested that the combination of low concentrations of salt ions with charged biopolymers weakens the inter- or intra-molecular repulsive force, thereby increasing the solubility of polysaccharides and peptides in solvents. According to Li et al. (2018) [40], there is a critical salt concentration where the coacervates are disassembled, and the concentration of salt at which the complexes is dissolved is known as the salt resistance. An increase in salt concentration leads to an increase in particle size of the polymer, which could cause instability of the composite system. Another explanation by Mangelsdorf and White (1998) [41] stated that a tight layer of colloidal particles could adsorb salt ions in a low salt concentration; when the adsorption site of the tight layer reaches saturation, the continually increased concentration of salt ions will cause ions with opposite charges in the diffusion layer to be squeezed into the tight layer, where the colloidal particles are compressed by the electric double layer to lose stability.

The surface charge of nisin/C6-Cc complexes also decreased with increasing ion concentration in Figure 5B. A possible explanation might be that there are more Na^+^ and Cl^−^ in the high concentration salt ion system, combined with the negative charge on C6-Cc and the positive charge on nisin, respectively. Therefore, the surface charge of the complex system showed a downward trend. Moreover, the nisin/C6-Cc complexes had a smaller particle size (all within 750 nm) when the ionic strength range was 0–100 mM. According to our recent study [21], the complex coacervation reaction of nisin and C6-Cc cannot be completely inhibited even at an ion concentration of 300 mM, which indicated that the nisin/C6-Cc complexes can resist the shielding effect of a high concentration of salt ions. Xiong et al. (2018) [42] compared the maximum turbidity values of ovalbumin/chitosan complexes (OVA/CMC) 0.7 and OVA/CMC 1.2 at the same ionic strength by an acidification turbidity curve and proposed that after different degrees of substitution, CMC with a high charge density had strong resistance to salt ion shielding, which was also consistent with this study. Moreover, Weinbreck et al. (2003) [43] found that whey protein and gum arabic could not co-coagulate when the salt ion concentration was greater than 50 mM. In contrast, for strong polyanionic polysaccharides such as carrageenan, the complex coacervation of whey protein and carrageenan can be completely inhibited when the ion concentration is as high as 400 mM [43]. Compared with these results, the nisin/C6-Cc complexes have a better salt ion stability with a smaller particle size in the range of 0–100 mM, which is beneficial to broaden the application range of nisin in the food preservation field.

### 3.5. Storage Stability of Nisin/C6-Cc Complexes

To evaluate the protective performance of the complexes, the storage stability of the nisin/C6-Cc complexes was then discussed. In Figure 5C, the particle size of the complexes increased with the extension of storage time at 25 °C, and the growth rate of the particle size is extremely slow within the storage time of 0–14 days. After 14 days, the particle size increased from 625.8 nm to 705.4 nm and reached 888.6 nm in the end. Moreover, the Zeta potential of nisin/C6-Cc complexes decreased slowly from −26.2 mV to −17.5 mV at 25 °C, which indicated that slight aggregation may occur in the system. These results revealed that nisin/C6-Cc complexes were stable within 14 days when stored at 25 °C. Figure 5D further showed the changes in particle size, Zeta potential, and PDI values of nisin/C6-Cc complexes at the 4 °C storage condition. The particle size of the nisin/C6-Cc complexes increases over time, which has a similar trend to that at 25 °C. However, the particle size of the system started to change significantly from 613.1 nm to 728.2 nm until the 21st day at 4 °C, where the occurrence time is remarkably delayed. In addition, the Zeta potential of nisin/C6-Cc hardly changed during storage. The storage stability of nisin/C6-Cc complexes as a preservative could avoid the problem of activity decay of free nisin during refrigerated storage. The results indicate that the structure and activity stability of the complex in storage are dominated by temperature-regulated intermolecular strength. At 4 °C, low temperatures could enhance the hydrogen bonding and electrostatic interaction between C6-Cc and nisin, where the main chain conformation of C6-Cc glucan was rigid, the crosslinking network was dense, and the thermal motion of particles was weak. Therefore, the particle size and Zeta potential changed smoothly within 21 days. While at 25 °C, the molecular thermal motion was intensified, some hydrogen bonds were broken, leading to a weakened electrostatic interaction; the surface charge of the complex decreased (where the Zeta potential dropped from −26.2 mV to −17.5 mV); and the particle repulsion was insufficient, resulting in secondary aggregation (28-day size up to 888.6 nm). Therefore, the above results showed that the low-temperature environment was more conducive to the storage of nisin/C6-Cc, the 1:8 ratio was the most stable cross-linked network due to the positive charge balance between C6-Cc carboxyl group and nisin, and storage at 4 °C could effectively prolong the shelf life of nisin/C6-Cc complexes. This is also consistent with the antibacterial activity results of nisin /C6-Cc complexes.

**Figure 6:**
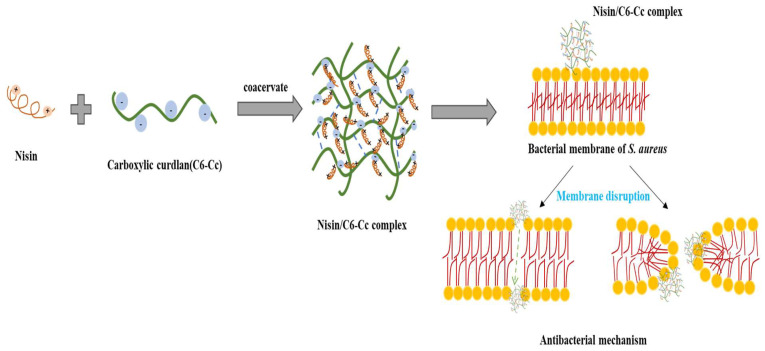
Antibacterial mechanism of nisin/C6-Cc complex.

Overall, this evidence indicates that nisin/C6-Cc complexes have high tolerance and stability for acid–base stress and possess good thermo-stability, thus identifying them as a promising preservative candidate for heat- and acid-treated food processing. Combined with the potential antibacterial mechanism presented in Figure 6, it can be speculated that nisin/C6-Cc complexes have a stable covalent interaction, a compact advanced structure, and a relative wide range of tolerance to pH and heat. The advantages of nisin/C6-Cc complexes include higher structural stability, lower cost, and ease of synthesis, as well as their resulting higher production efficiency. Moreover, the amphiphilic structure of nisin in coacervated complexes exhibit high antibacterial efficiency, which increase its application value in the food industry. For instance, the characteristics of the complexes could make it widely adaptable in acidic beverages at pH 1–4. For canned, sterilized dairy products and other foods that need to be treated at 121 °C, the complexes could still maintain a significant antibacterial effect after extreme temperature treatment, breaking through the bottleneck of free nisin inactivation at high temperatures. In pickles and low-salt meat products with salt concentrations of 0–100 mM, its stable particle size (<750 nm) and constant MIC value (0.71 mg/mL) can specifically inhibit foodborne pathogens such as *S. aureus* and adapt to a high-salt processing environment. In refrigerated foods such as chilled meat and low-temperature yogurt, the stable physicochemical and antibacterial properties of the complex within 28 days can effectively prolong the shelf life of the product and reduce the risk of deterioration during refrigerated storage.

In summary, nisin/C6-Cc complexes are expected to become an ideal candidate preservative in the field of food biological preservation due to their comprehensive advantages of ‘acid resistance, heat resistance, salt resistance and storage resistance’. This provides a new technical path for the safe preservation of acidic, high-temperature processed, high-salt, and refrigerated foods and has important industrial application potential. In the future, its suitability in composite food matrixes and large-scale application processes can be further explored to promote the wide application of natural antimicrobial peptides in the food industry.

## 4. Conclusions

In this study, the nisin/C6-Cc complexes showed remarkable antibacterial stability under various conditions, where the pH fluctuation range was within 1 to 4; the salt ion concentration was from 0 to 200 mM; and stability was found in a wider range of temperatures, at 25–121 °C. Nisin/C6-Cc complexes formed at the C6-Cc to nisin mass ratio of 1:8 (*w*/*w*) were stable under conditions with pH < 5 and 121 °C and could maintain their physicochemical properties for 28 days in storage at 4 °C. Therefore, this research provides more specific technical support for nisin from laboratory to industrial application.

## Figures and Tables

**Table 1:** Effects of temperature on MIC values of nisin/C6-Cc complexes (MIC, mg/mL).

Molar Ratio of Samples (*w*/*w*)	Temperature (°C)					
	25	40	60	80	100	121
1:4	0.83	0.83	0.83	0.83	0.83	0.83
1:8	0.71	0.71	0.71	0.71	0.71	0.71
1:14	0.51	1.03	1.03	1.03	1.03	1.03

**Table 2:** Effect of storage conditions on MIC values of nisin/C6-Cc complexes (MIC, mg/mL).

Molar Ratio of Samples (*w*/*w*)	Storage Conditions		
	0 Day	30 Days, 25 °C	30 Days, 4 °C
1:4	0.83	0.83	0.83
1:8	0.71	0.71	0.71
1:14	0.51	1.03	1.03

**Table 3:** Effect of pH values on MIC values of nisin/C6-Cc complexes (MIC, mg/mL).

Samples	Nisin	C6-Cc	pH of Nisin/C6-Cc						
			1	2	3	4	5	6	7
MIC	0.31	—	0.71	0.71	0.71	0.71	1.43	1.43	1.43
Samples			Ionic strength (mM)						
			0	50	100	150		200	
MIC			0.71	0.71	0.71	0.71		0.71	

“—” means there is no antibacterial activity.

## Data Availability

The original contributions presented in this study are included in the article. Further inquiries can be directed to the corresponding author.
